# *QuickStats:* Mental Health Treatment Trends* Among Adults Aged ≥18 Years, by Age Group — United States, 2019–2023^^†^^

**DOI:** 10.15585/mmwr.mm7350a5

**Published:** 2024-12-19

**Figure:**
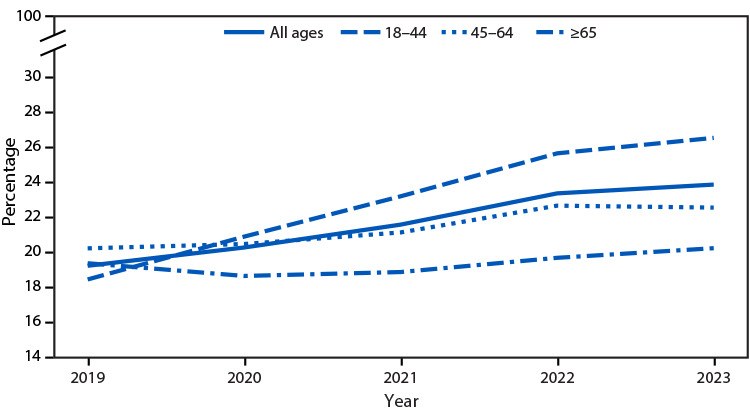
From 2019 to 2023, the percentage of adults who had received any mental health treatment during the past 12 months increased from 19.2% to 23.9%. This pattern was similar among adults aged 18–44 and 45–64 years. No significant change was observed among adults aged ≥65 years.

**Supplementary Table:** https://stacks.cdc.gov/view/cdc/174552

**Source:** National Center for Health Statistics, National Health Interview Survey, 2019–2023. https://www.cdc.gov/nchs/nhis.htm

For more information on this topic, CDC recommends the following link: https://www.cdc.gov/mental-health/.

